# Heating Efficiency of Triple Vortex State Cylindrical Magnetic Nanoparticles

**DOI:** 10.1186/s11671-019-3169-6

**Published:** 2019-12-16

**Authors:** De Wei Wong, Wei Liang Gan, Yuan Kai Teo, Wen Siang Lew

**Affiliations:** 1 School of Physical and Mathematical Sciences, Nanyang Technological University, 21 Nanyang Link, Nanyang, 637371 Singapore; 2 School of Biological Sciences, Nanyang Technological University, 60 Nanyang Drive, Nanyang, 637551 Singapore

**Keywords:** Magnetic nanoparticles, Magnetic hyperthermia, Specific absorption rate, HeLa cells

## Abstract

A well-established method for treating cancerous tumors is magnetic hyperthermia, which uses localized heat generated by the relaxation mechanism of magnetic nanoparticles (MNPs) in a high-frequency alternating magnetic field. In this work, we investigate the heating efficiency of cylindrical NiFe MNPs, fabricated by template-assisted pulsed electrodeposition combined with differential chemical etching. The cylindrical geometry of the MNP enables the formation of the triple vortex state, which increases the heat generation efficiency by four times. Using time-dependent calorimetric measurements, the specific absorption rate (SAR) of the MNPs was determined and compared with the numerical calculations from micromagnetic simulations and vibrating sample magnetometer measurements. The magnetization reversal of high aspect ratios MNPs showed higher remanent magnetization and low-field susceptibility leading to higher hysteresis losses, which was reflected in higher experimental and theoretical SAR values. The SAR dependence on magnetic field strength exhibited small SAR values at low magnetic fields and saturates at high magnetic fields, which is correlated to the coercive field of the MNPs and a characteristic feature of ferromagnetic MNPs. The optimization of cylindrical NiFe MNPs will play a pivotal role in producing high heating performance and biocompatible magnetic hyperthermia agents.

## Introduction

The applications for magnetic nanoparticles (MNPs) have been extensively researched in biomedical fields, such as magneto-mechanical cell destruction [1–4], magnetic resonance imaging [5–7], drug delivery [8–10], and magnetic hyperthermia [11–14], to compensate for the drawbacks of current diagnosis and therapy methods. The greatest advantage of MNPs is that they can be controlled remotely by an external magnetic field. The resultant magnetic response can be in the form of heat dissipation or magnetic torque, which is dependent on the applied magnetic field configuration and magnetization dynamics of the MNPs [15].

However, different biomedical applications require specific rotation mechanisms in diverse magnetic field configurations. Bio-sensors for cancer bio-markers use magnetic spectroscopy of the MNPs’ Brownian motion to measure the bound fraction and relaxation times of the MNPs within seconds [16]. In magnetic particle imaging for quantifying MNP concentrations, it requires Néel relaxation of the MNPs, while Brownian relaxation, caused by MNPs size distributions, should be minimized [17]. The two mechanisms that exist for the MNPs relaxation processes are Néel and Brownian relaxation, which results in either heat dissipation or spatial rotation of the MNPs. Néel relaxation is correlated to the re-orientation of the MNP magnetic moment to the magnetic field, while Brownian relaxation is correlated to the spatial rotation of the MNP [18–20].

Néel (*t*_*N*_) and Brownian (*t*_*B*_) relaxation time are given by:

$$ {t}_N={t}_0{e}^{\frac{KV}{k_BT}}\; and\;{t}_B=\frac{3\eta\;V}{k_BT} $$

where *η* is the viscosity coefficient, *t*_*0*_ is the inverse attempt frequency, *K* is the magnetic anisotropy constant, *V* is the volume of MNPs, *k*_*B*_ is the Boltzmann constant, and *T* is the temperature. In principle, the faster mechanism dominates, but both Néel and Brownian mechanisms can occur concurrently, coupled through heat dissipation and magnetic torque [21]. The effective relaxation time (*t*_*eff*_) is given by:

$$ \frac{1}{t_{eff}}=\frac{1}{t_B}+\frac{1}{t_N} $$

In smaller MNPs, the dominant mechanism is Néel relaxation, while for larger MNPs it is Brownian relaxation. In Néel relaxation, the MNP magnetization changes direction due to the reconfiguration of its magnetic moment and is dependent on the MNP size and the temperature. While in Brownian relaxation, the MNPs undergo a spatial rotation and are dependent on external conditions, such as viscosity and chemical binding [22–24]. Therefore, it is important to understand the contributions of these magnetic relaxation mechanisms in order to tune and adapt the design of the MNPs to obtain the optimal heat generation for magnetic hyperthermia or magnetic torque for magneto-actuated cell death.

Magnetic hyperthermia is a well-established cancer treatment technique which employs the use of localized heating by MNPs under a high-frequency alternating magnetic field, to induce cancer cell apoptosis and tumor regression [3, 25–27]. In an alternating magnetic field, the heat dissipated by the MNPs in one magnetic field cycle equals to the area of the hysteresis loop *A*, given by:

$$ A={\int}_{-{H}_{\mathrm{max}}}^{+{H}_{\mathrm{max}}}\;{\mu}_0\;M(H)\; dH $$

where *M* is the magnetization of MNPs, under an alternating magnetic field with frequency *f* and amplitude *μ*_*0*_*H*_*max*_ [28–30]. To maintain a low MNPs dose and short treatment duration in magnetic hyperthermia, the MNPs heating efficiency must be maximized. The measurement of MNPs heating performance is referred to as specific absorption rate (SAR), which is given by the heat dissipated per unit of mass of MNPs (Wg^− 1^):

$$ \mathrm{SAR}=\frac{A\;f}{\rho } $$

where *ρ* is the density of MNPs.

The efficiency of heat dissipation of MNPs can be experimentally measured in terms of SAR, which is the energy dissipated per unit of mass of MNPs (Wg^− 1^), and is given by:

$$ \mathrm{SAR}=C\frac{\varDelta T}{\varDelta t}\frac{1}{m_{\mathrm{MNP}}} $$

where *C* is the specific heat of the medium (*C*_water_ = 4.18 Jg^− 1^ °C^− 1^), *ΔT*/*Δt* is the initial slope of the time against temperature graph, and *m*_MNP_ is the mass of the MNPs. However, SAR values are not fully representative of the heating efficiency of MNPs as heat dissipation is also influenced by frequency *f* and magnetic field strength *H*. Hence, effective specific absorption rate or intrinsic loss power (ILP) is used to characterize the MNPs heating efficiency, given by:

$$ \mathrm{ILP}=\frac{\mathrm{SAR}}{H^2f} $$

In cylindrical NiFe MNPs, a triple vortex state is formed, in which clockwise and anti-clockwise vortices are connected at the center of the MNP via a third vortex core, resulting in a three-dimensional magnetization configuration. The theoretical heat dissipation from the MNPs for magnetic hyperthermia applications was calculated from the simulated hysteresis loops and vibrating sample magnetometer measurements. Using time-dependent calorimetric measurements, the specific absorption rate and intrinsic loss power of the MNPs were determined and compared with the numerical calculations.

## Methods

### Fabrication of Magnetic Nanoparticles

Template-assisted pulsed electrodeposition with differential chemical etching is a simple and inexpensive fabrication method to produce MNPs of various compositions, Ni, Fe, or Co. Ni_80_Fe_20_, Permalloy, is a ferromagnetic material that displays exceptional magnetic properties such as high permeability, low coercivity, and near-zero magnetostriction. The fabrication of cylindrical MNPs starts by growing compositionally modulated cylindrical NiFe nanowires using anodic aluminum oxide (AAO) template-assisted pulsed electrodeposition in an electrolyte bath consisting of NiSO_4_, FeSO_4_, and H_3_BO_3_ [31–35]. Subsequently, the nanowires were released by dissolving the AAO template in NaOH. Finally, the Fe-rich regions in the nanowires were chemically etched by diluting HNO_3_ to form the MNPs. The diameter of the MNPs was determined by the AAO template pore size, while the length was controlled by the high-potential pulse *V*_*H*_ duration Additional file 1.

### Cell Viability

HeLa cells were seeded into 12-well microtiter plate at 8 × 10^4^ cells/well and incubated in Dulbecco’s Modified Eagle’s medium supplemented with 4.5 g/L glucose, 2 mM L-glutamine, 10% fetal bovine serum, and 1% penicillin/streptomycin in a humidified atmosphere at 37 °C and 5% CO_2_. The cell viability was determined using PrestoBlue, a permeable resazurin-based cell viability reagent, which uses the reducing power of viable cells to quantitatively measure cell proliferation. HeLa cells treated with 0.1 mg/ml of MNPs were incubated with the PrestoBlue reagent at 37 °C and 5% CO_2_ for 2 h. The absorbance values at 570 nm and 600 nm were measured by Tecan Infinite M200 PRO Microplate Reader. The cell viability was expressed as a percentage relative to the cells unexposed to the MNPs. Each experiment was performed in quadruplicate sets of experimental and control assays.

### Statistical Analysis

The results were represented as the mean ± standard deviation (SD). Statistical significance was analyzed using one-way analysis of variance (ANOVA) with OriginPro, OriginLab. A *p* value of < 0.05 was considered to be statistically significant.

### Micromagnetic Simulations

The magnetization configurations of the MNPs were investigated using a GPU-accelerated micromagnetic simulation program, MuMax3, to solve the Landau–Lifshitz–Gilbert (LLG) equation in three dimensions [36]. These micromagnetic simulations provided insights into the magnetization configurations of the MNP at the microscopic level, which showed the correlation between analytical models and observations from experimental results. The total energy of a system is described by:

$$ {E}_{\mathrm{Total}}={E}_{\mathrm{Exchange}}+{E}_{\mathrm{Anisotropy}}+{E}_{\mathrm{Zeeman}}+{E}_{\mathrm{Dipolar}}=-\int {\mu}_0{H}_{eff}(r)\cdot M(r){d}^3r $$

where ${H}_{eff}=-\frac{1}{\mu_0}{\nabla}_ME$. The Landau–Lifshitz–Gilbert (LLG) equation describes the precession of magnetization ***M*** in an effective magnetic field ***H***_***eff***_ with damping *α*.

$$ \frac{dM(r)}{dt}=-\gamma M(r)\times {H}_{eff}(r)-\frac{\overline{\alpha}}{M_s}M(r)\times \left(M(r)\times {H}_{eff}(r)\right) $$

where *γM*(*r*) × *H*_*eff*_(*r*) is the precession of *M*(*r*) in a local field *H*_*eff*_(*r*) and $\frac{\overline{\alpha}}{M_s}M(r)\times \left(M(r)\times {H}_{eff}(r)\right)$ is the empirical damping term. The material parameters for Permalloy Ni_80_Fe_20_ were used: saturation magnetization *M*_*s*_ of 860 × 10^3^ A/m, exchange stiffness constant *A*_*ex*_ of 1.3 × 10^− 11^ J/m, zero magneto-crystalline anisotropy *k* = 0, and Gilbert damping constant *α* of 0.01. A cell size of 5 nm × 5 nm × 5 nm was used for all simulations, which is sufficiently small as compared with the exchange length.

### Experimental Setup for Magnetic Hyperthermia

SAR was experimentally obtained from time-dependent calorimetric measurements by exposing the MNPs to an alternating magnetic field generated by a high-frequency induction heater. MNPs in aqueous suspension with concentrations of 0.05–0.1 mg/ml were poured into a falcon tube, which was insulated by styrofoam and surrounded by the induction coils. The temperature of the coils was maintained at 28.0 ± 0.5 °C by a water recirculating chiller. The initial temperature of the suspension was maintained at 28.0 ± 0.5 °C for 1 min to eliminate any heat contributions from the induction coils. An alternating magnetic field range of 15.9 to 47.8 kAm^− 1^ and fixed frequency of 360 kHz were applied, within the criterion for clinical magnetic hyperthermia.

## Results and Discussion

### Characterization of Magnetic Nanoparticles

**Fig. 1:**
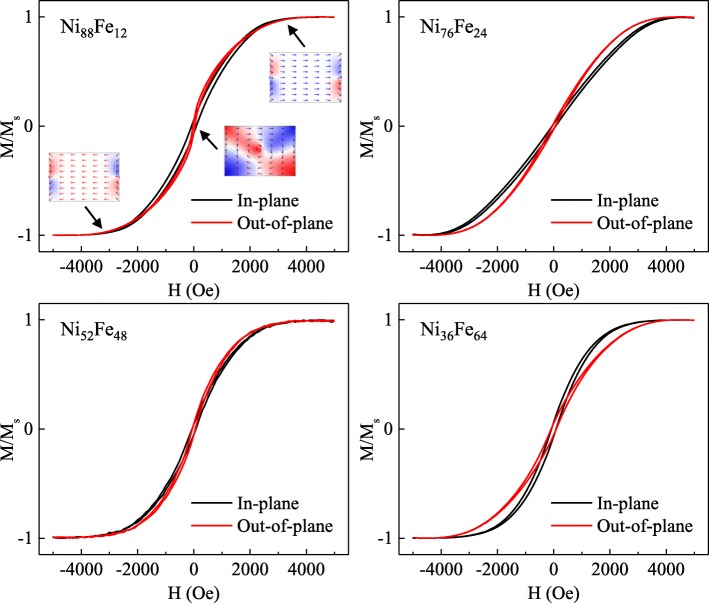
Normalized hysteresis loops of NiFe MNPs for Ni_88_Fe_12_, Ni_76_Fe_24_, Ni_52_Fe_48_, and Ni_36_Fe_64_ measured in the in-plane and out-of-plane directions. Insets show the magnetization configurations of the MNP at various magnetic field strengths

The composition of the fabricated cylindrical NiFe MNPs is determined by *V*_*H*_ or the electrolyte composition. To show the large degree of control in the MNPs composition, various compositions of MNPs have fabricated (Ni_88_Fe_12_, Ni_76_Fe_24_, Ni_52_Fe_48_, and Ni_36_Fe_64_) and verified by energy-dispersive X-ray spectroscopy (EDX). Figure 1 shows the normalized hysteresis loop obtained by vibrating sample magnetometer (VSM) measurements for the NiFe MNPs with various compositions. The magnetic field is increased to a value that is sufficient to overcome the effective magnetic anisotropy such that the magnetization reaches saturation. The squareness ratio SQR is a basic measurement of how square the hysteresis loop is, given by:

$$ \mathrm{SQR}=\frac{M_r}{M_s} $$

The values of coercivity *H*_*c*_ and squareness SQR = *M*_*r*_/*M*_*s*_ for in-plane and out-of-plane applied magnetic fields are tabulated in Table 1. In general, the trend of in-plane *H*_*c*_ is higher than the out-of-plane *H*_*c*_ for Ni-rich MNPs (Ni_88_Fe_12_, Ni_76_Fe_24_, and Ni_52_Fe_48_), but reversed for Fe-rich MNPs (Ni_36_Fe_64_), which is in agreement with the previous studies on anomalous co-deposition of NiFe nanowires [37].

**Table 1:** Coercivity *H*_*c*_ and squareness SQR = *M*_*r*_/*M*_*s*_ values of NiFe MNPs for Ni_88_Fe_12_, Ni_76_Fe_24_, Ni_52_Fe_48_, and Ni_36_Fe_64_

Ni:Fe ratio	In-plane *H*_*c*_ (Oe)	Out-of-plane *H*_*c*_ (Oe)	In-plane SQR	Out-of-plane SQR
Ni_88_Fe_12_	84.5	33.8	0.060	0.054
Ni_76_Fe_24_	62.7	54.0	0.023	0.027
Ni_52_Fe_48_	68.6	51.4	0.048	0.049
Ni_36_Fe_64_	80.7	91.1	0.064	0.054

### Biocompatible Surface Coating

**Fig. 2:**
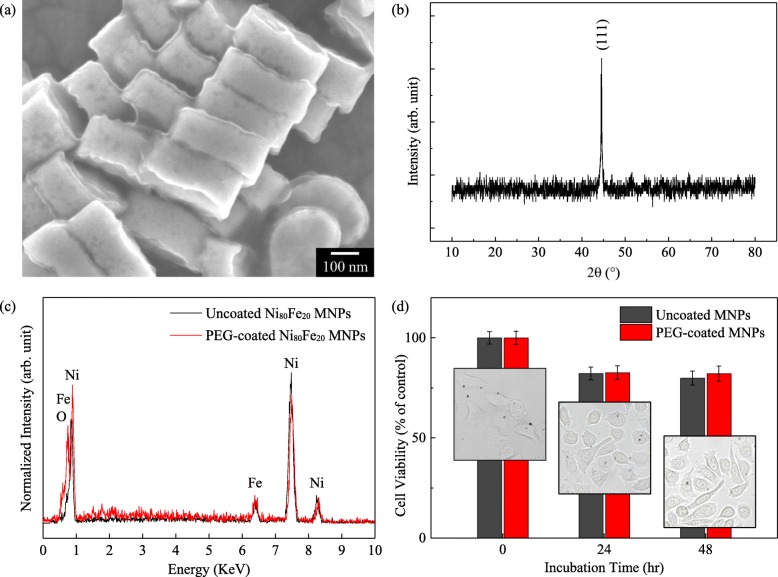
**a** SEM image of NiFe MNPs with PEG coating. **b** XRD pattern for NiFe MNPs with PEG coating. **c** EDX pattern for NiFe MNPs with and without PEG coating. **d** Cell viability with displayed images of HeLa cells after incubation with MNPs, over a period of 0–48 h

NiFe MNPs tend to aggregate due to the effects of strong dipole interactions between neighboring MNPs. Therefore, the surface modification of the MNPs using biocompatible and biodegradable polymer [38, 39], such as chitosan [40–42], polyvinyl alcohol [43–45], oleic acid [46–48], dextran [49, 50], and most commonly polyethylene glycol (PEG) [51–56], has been proposed. PEG is a hydrophilic polymer that has been widely used for improving blood circulation of liposomes and MNPs [57–60]. To disperse the cylindrical NiFe MNPs into water, a biocompatible 5000 g mol^− 1^ PEG was used as a stabilizer [61]. The scanning electron microscopy (SEM) image shows the formation of an oxide shell around the MNPs, shown in Fig. 2a. This oxide shell prevents oxidation of the magnetic materials in the MNPs. Previous research works on FeCo MNPs and Fe MNPs have shown severe oxidation from just exposure to the atmosphere [61, 62].

The X-ray diffraction (XRD) pattern peak was mainly indexed at the (111) crystal planes which corresponds to the face-centered cubic (fcc) structure of bulk NiFe, as shown in Fig. 2b. This indicates that the MNPs were electrodeposited with a preferred orientation of (111), which is also evident in NiFe nanowires fabricated by electrodeposition or sputtering [63, 64]. In addition, there was an absence of diffraction peaks corresponding to spinel oxide ((NiFe)_3_O_4_), which results from the formation of oxide phases due to the high concentration of Fe [65]. The high crystallinity of the NiFe MNPs led to negligible surface spin canting and hence retained the high saturation magnetization and small coercivity of the MNPs. Further characterizations for the PEG-coated NiFe MNPs were conducted using EDX measurement. As shown in Fig. 2c, mainly of Ni and Fe elements were detected, with the presence of a small percent of O element, an indication of the oxide shell formed around the MNPs.

From Fig. 2d, the cell viability of the HeLa cells exposed to uncoated and PEG-coated NiFe MNPs after 24 h is 82.2% and 82.6%, respectively. After 48 h, the cell viabilities decreased slightly to 79.9% and 82.1%, displaying slightly higher biocompatibility for PEG-coated MNPs. NiFe MNPs without any shells are toxic to mammalian cells and will affect cell viability. The PEG coating was highly biocompatible and can decrease the cytotoxicity and internalization of MNPs into the cells due to endocytosis [66, 67]. The cytotoxicity of the cylindrical NiFe MNPs to HeLa cells is comparable with other commercially available ferromagnetic NPs used in magnetic hyperthermia research [68].

### Magnetization Dynamics

**Fig. 3:**
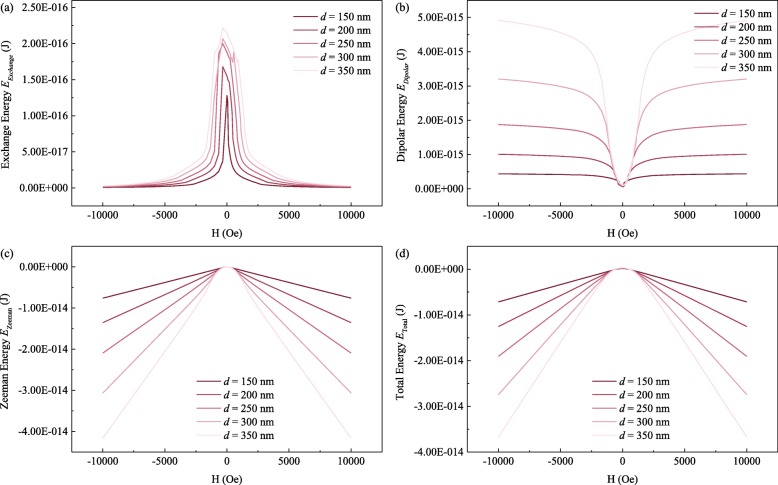
The plot of (**a**) exchange energy, (**b**) dipolar energy, (**c**) Zeeman energy, and (**d**) total energy against applied magnetic field *H*

The composition of the MNPs was kept at Permalloy Ni_80_Fe_20_, while the length (*l*) and diameter (*d*) of the MNPs were varied. The exchange energy, demagnetization, or dipolar energy and Zeeman energy contributions to the total energy of the MNP are plotted as a function of applied magnetic field *H* along the MNP long axis in Fig. 3a–d, respectively. The MNP was first saturated by a strong magnetic field parallel to its long axis. At large magnetic fields, the Zeeman energy contribution predominates and the spins are mostly aligned in the magnetic field direction. This parallel arrangement of the spins to the field minimizes the exchange energy contribution to the total magnetic energy. As the applied magnetic field is reduced, a clockwise and an anti-clockwise vortex nucleation occur at the ends of the MNP, which progress towards the center of the MNP, leading to a gradual reduction of the parallel magnetization component that causes a drop in the Zeeman contribution, while other contributions become increasingly significant. The magnetization of the MNP tries to minimize the stray field, and thus reducing its demagnetization energy. At sufficiently low magnetic fields, the triple vortex state is formed, which is a stable magnetization configuration, with total energy kept at a minimum. As the magnetic field reverses, the sharp drop in exchange energy corresponds to the abrupt splitting of the two vortices.

MNPs with different lengths (*l*) were found to have significantly different magnetization configurations. At lengths *l* below 100 nm, only a single vortex was nucleated, which is an in-plane and closed flux domain structure, owing to the interaction between the magnetostatic energy and exchange energy. For *l* above 100 nm, a pair of anti-clockwise and clockwise vortex cores at the ends of the MNP were nucleated—double vortex state. When the magnetic field decreases, one of the vortex is annihilated, collapsing into the single vortex state. However, at *l* above 300 nm, there is no annihilation of vortex at low fields, instead an additional third vortex core was nucleated on the curved surface of the MNP—triple vortex state.

### Calorimetric Measurements

**Fig. 4:**
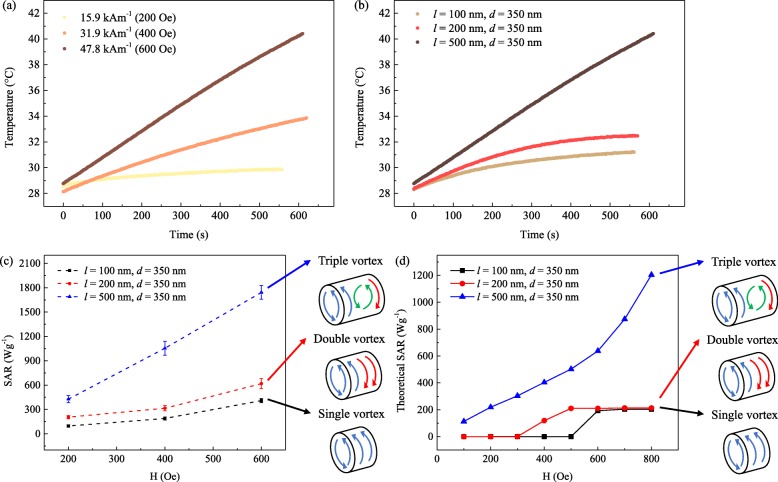
**a** Temperature-time curves of NiFe MNPs, with *l* = 500 nm and *d* = 350 nm, for increasing magnetic field strength from 15.9 to 47.8 kAm^− 1^ (200 to 600 Oe). **b** Temperature-time curves of NiFe MNPs, with *d* = 350 nm. The magnetic field strength of 47.8 kAm^− 1^ (600 Oe), while the MNP length *l* increased from to 500 nm. **c** Tabulated SAR values for NiFe MNPs, with *l* = 100–500 nm and *d* = 350 nm under a magnetic field strength of 15.9 to 47.8 kAm^− 1^ (200 to 600 Oe). **d** Theoretical SAR values for NiFe MNPs, with *l* = 100–500 nm and *d* = 350 nm

The Ni_80_Fe_20_ MNPs, with *l* = 500 nm and *d* = 350 nm, were exposed to an alternating magnetic field of 15.9 to 47.8 kAm^−1^ (200 to 600 Oe), and the temperature-time curve is displayed in Fig. 4a. As characterized by the SAR equation, the SAR values were calculated to be 427 Wg^− 1^, 1054 Wg^− 1^, and 1742 Wg^− 1^, for 15.9 kAm^− 1^, 31.9 kAm^− 1^, and 47.8 kAm^− 1^, respectively. As predicted, the larger the magnetic field strength, the greater the SAR value, i.e., the SAR value was proportional to the magnetic field strength. Therefore, ILP was obtained to give a better evaluation of the heating efficiency of the MNPs for magnetic hyperthermia. As characterized by the ILP equation, the ILP values were calculated to be 4.69 nHm^2^kg^− 1^, 2.88 nHm^2^kg^− 1^, and 2.12 nHm^2^kg^− 1^, for 15.9 kAm^− 1^, 31.9 kAm^− 1^, and 47.8 kAm^− 1^ at 360 kHz, respectively.

Next, NiFe MNPs, with *d* = 350 nm and *l* = 100–500 nm, were exposed to an alternating magnetic field of 47.8 kAm^− 1^ (600 Oe), and the temperature-time curve is displayed in Fig. 4b. As characterized by the SAR equation, the SAR values were calculated to be 409 Wg^− 1^, 618 Wg^− 1^, and 1742 Wg^− 1^, for *l* = 100 nm, 200 nm, and 500 nm at 47.8 kAm^− 1^ and 360 kHz, respectively. As characterized by the ILP equation, the ILP values were calculated to be 0.50 nHm^2^kg^− 1^, 0.75 nHm^2^kg^− 1^, and 2.12 nHm^2^kg^− 1^ for *l* = 100 nm, 200 nm, and 500 nm at 47.8 kAm^− 1^ and 360 kHz, respectively.

MNPs with *l* = 500 nm had far greater heating efficiency than MNPs with *l* = 100 nm and 200 nm, leading to a more significant temperature rise. The highest SAR value of MNPs with *l* = 500 nm was 1742 Wg^− 1^ at 47.8 kAm^− 1^ and 360 kHz. For comparison, the SAR values for magnetic field of 15.9 to 31.9 kAm^− 1^ (200 to 400 Oe) and MNPs with *d* = 350 nm and *l* = 100–500 nm were tabulated in Fig. 4c. Under the same conditions, the SAR and ILP values of MNPs with *l* = 500 nm were four times higher than those with MNPs of smaller *l*. From micromagnetic simulations, it was observed that as *l* increases to > 300 nm, the magnetization reversal process of the MNP changed from a double vortex state to triple vortex state. At *l* < 300 nm, only a single vortex state or double vortex state was observed. The remanent magnetization *M*_*r*_ of the MNP was significantly higher for the triple vortex state as compared with the single or double vortex state.

For single domain MNPs, the theoretical model to calculate the dynamic hysteresis loop has been proposed by Carrey et al. [69] For multi-domains MNPs, the use of micromagnetic simulations to obtain static hysteresis loop for calculation was reasonable for MNPs with large sizes, above the critical size for superparamagnetism, as the switching time of the magnetization is in the order of 10^− 9^ s. Since the switching time of magnetic hyperthermia is in the order ~ 10^− 6^ s, the large MNPs are able to keep up with the alternating magnetic field. The area of hysteresis loops obtained from micromagnetic simulations of cylindrical NiFe MNPs and VSM measurements was used to theoretically calculate the SAR values and tabulated in Fig. 4d.

The SAR values of MNPs with *l* = 100 nm and 200 nm displayed a small SAR value at low magnetic fields below *H*_*c*_ and sharply increased until it reaches a saturation at high magnetic fields, which is a characteristic of the ferromagnetic regime. In contrast, the magnetic field dependence of the SAR value of *l* = 500 nm MNPs, with the triple vortex state, followed a non-linear relationship with SAR values that were ~ 6 times greater. The high remanent magnetization *M*_*r*_ of the triple vortex state in the *l* = 500 nm MNPs was evident in the non-zero SAR values at low magnetic fields. The comparison between calorimetric measurements (Fig. 4c) and numerical calculations (Fig. 4d) indicates a qualitative and quantitative agreement on the features of MNPs in the ferromagnetic regime, displaying small SAR values at low magnetic fields and saturation at high magnetic fields which was correlated to the *H*_*c*_ of the MNPs.

**Fig. 5:**
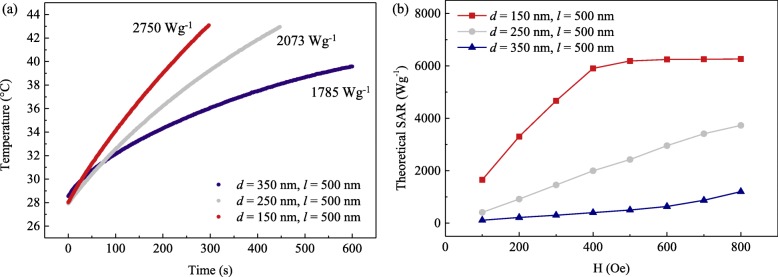
**a** Temperature-time curves of NiFe MNPs under a magnetic field strength of 47.8 kAm^− 1^ (600 Oe) and frequency of 360 kHz, while the MNP length *d* increased from 150 to 350 nm. **b** Theoretical SAR values for NiFe MNPs, with *d* = 150–350 nm and *l* = 500 nm

The heat dissipation of NiFe MNPs with triple vortex states was compared for *d* = 150–350 nm, under an alternating magnetic field of 47.8 kAm^− 1^ (600 Oe), and the temperature-time curve is displayed in Fig. 5a. The SAR and ILP values were calculated to be 1785 Wg^− 1^, 2073 Wg^− 1^, and 2750 Wg^− 1^ and 2.17 nHm^2^kg^− 1^, 2.52 nHm^2^kg^− 1^, and 3.34 nHm^2^kg^− 1^, for *d* = 350 nm, 250 nm, and 150 nm, respectively. The MNPs with *d* = 150 nm and 250 nm were able to reach the optimal therapeutic temperature of 43 °C in 4.92 min and 7.45 min at concentration of 0.1 mg/ml. Comparing MNPs with different aspect ratios, it was observed that the heating efficiency of *d* = 150 nm MNPs was 1.54 times greater than *d* = 350 nm MNPs. This was because MNPs with *d* = 150 nm possessed the highest low-field susceptibility and *M*_*r*_. Therefore, the SAR value was closely correlated to the magnetization reversal process of the MNPs with both variations in *l* and *d*.

From micromagnetic simulations, it can be observed that the area of hysteresis *A* evolves significantly with the diameter (*d*) of the MNP. Therefore, the SAR value of the *d* = 150 nm MNPs increases so rapidly and saturates at a maximum SAR value of 6263 Wg^− 1^. The numerical calculations showed that the MNPs with higher aspect ratios have higher hysteresis losses, resulting in higher theoretical SAR values, as shown in Fig. 5b. The comparison between calorimetric measurements (Fig. 5a) and numerical calculations (Fig. 5b) was in good qualitative agreement, but there were quantitative disagreements in the values of hysteresis losses. The mismatch between the experimental and theoretical values arose from the NiFe MNPs being non-superparamagnetic and possessed non-negligible remanent magnetization, leading to unwanted agglomeration due to strong magnetic dipole interactions between neighboring MNPs [70, 71]. Since hydrodynamic volume of MNPs is a component governing the Brownian motion, the extent of aggregation of the MNPs will determine the dominating relaxation mechanism, i.e., Néel or Brownian relaxation. Hence, an aggregated group of MNPs versus a single free MNP will greatly differ in SAR values. Furthermore, an alternating magnetic field can induce nano-columns or nano-chains formation which exhibit dissimilar Brownian relaxation mechanism and hence accounted for the discrepancy between the experimental and theoretical values [72–74].

## Conclusions

The high SAR values displayed by the cylindrical NiFe MNPs, comparable with iron oxide MNPs (IOMNPs) and superparamagnetic iron oxide nanoparticles (SPIONs) [28, 75], demonstrate the capability of these MNPs in heat dissipation under an alternating magnetic field. MNPs with the triple vortex state had far greater heating efficiency than MNPs with double or single vortex state, which have a SAR value of four times greater, attributed to the high *M*_*r*_ of the MNPs in the triple vortex state. Comparing MNPs with different aspect ratios, it was observed that the heating efficiency of *d* = 150 nm MNPs was 1.54 times greater than *d* = 350 nm MNPs due to a larger *M*_*r*_ and low-field susceptibility. Both calorimetric measurements and micromagnetic simulations showed the correlation between the magnetization reversal process and the higher hysteresis losses from *d* = 150 nm MNPs, resulting in higher experimental and theoretical SAR values. The easy control of the sizes of the MNPs and their magnetic properties indicate great potential for in vivo magnetic hyperthermia cancer therapy trials.

## Supplementary information

**Additional file 1.** Supplementary Material.

## Data Availability

The datasets generated during and/or analyzed during the current study are available from the corresponding author on reasonable request.

## Abbreviations

*d*: Diameter of magnetic nanoparticles
EDX: Energy-dispersive X-ray spectroscopy
*H*_*c*_: Coercivity
ILP: Intrinsic loss power
*l*: Length of magnetic nanoparticles
MNPs: Magnetic nanoparticles
*M*_*r*_: Remanent magnetization
PEG: Polyethylene glycol
SAR: Specific absorption rate
SEM: Scanning electron microscopy
SQR: Squareness ratio
*V*_*H*_: High-potential electrodeposition pulse
VSM: Vibrating sample magnetometer
XRD: X-ray diffraction spectroscopy
