# Supplementary material for: Heating Efficiency of Triple Vortex State Cylindrical Magnetic Nanoparticles
Source: Nanoscale Res Lett. 2019 Dec 16;14:376. doi: 10.1186/s11671-019-3169-6 (PMC6915247; doi:10.1186/s11671-019-3169-6)
Supplement: Supplementary file 1 — Additional file 1. Supplementary Material. [file 11671_2019_3169_MOESM1_ESM.docx]

**Additional file 1**

**Heating Efficiency of Triple Vortex State Cylindrical Magnetic Nanoparticles**

De Wei Wong,^1^ Wei Liang Gan,^1^ Yuan Kai Teo,^2^ and Wen Siang Lew^1,^ *

*^1^School of Physical and Mathematical Sciences, Nanyang Technological University
21 Nanyang Link, Singapore 637371*

*^2^School of Biological Sciences, Nanyang Technological University
60 Nanyang Drive, Singapore 637551*

**Corresponding author: wensiang@ntu.edu.sg*

Template-assisted pulsed electrodeposition with differential chemical etching is a simple and inexpensive fabrication method to produce MNPs of various compositions, Ni, Fe or Co. Ni_80_Fe_20_, Permalloy, is a ferromagnetic material that displays exceptional magnetic properties such as high permeability, low coercivity and near zero magnetostriction. The fabrication of cylindrical MNPs starts by growing compositionally modulated cylindrical NiFe nanowires using anodic aluminum oxide (AAO) template-assisted pulsed electrodeposition at 25 °C in an electrolyte consisting of 0.5M NiSO_4_, 0.01M FeSO_4_ and 0.5M H_3_BO_3_.(1-3) Subsequently, the AAO template was dissolved in 1M NaOH solution and the released nanowires were thoroughly rinsed with deionized water to de-alkalize. Finally, the Fe-rich regions in the nanowires were completely etched away in dilute HNO_3_ to form the MNPs. The dimensions of the MNPs were characterized by SEM images. The diameter of the MNPs (*d* = 150, 250 and 350 nm) was determined by the AAO template pore size, while the length (*l* = 75–500 nm) was controlled by the high potential pulse *V_H_* duration. An increase in the *V_H_* duration of 2 s to 40 s results in an increase in *l* from 75 nm to 500 nm, as shown in Figure S1.

**Figure S1** The graph of MNP length *l* vs high potential pulse *V_H_* duration.

The anomalous co-deposition phenomenon has been observed for the deposition of Fe in NiFe thin films and nanowires with a dependency on the applied electrodeposition potential, as shown in Figure S2.(4) In the deposition of Fe group metals, the less noble metal deposits preferentially as compared to the more noble ones due to anomalous co-deposition effect.(4) At lower potentials such as −0.9V, the proportion of Fe in the deposited alloy can almost be equal to the amount of Ni despite Fe ions being much scarcer than Ni ions.(5) However, with the increase of negative potential from −0.9 V to −1.4 V, the effect of anomalous co-deposition peaks at −1.3V and subsequently reduces till the co-deposition of NiFe alloys becomes non-anomalous.(6) The side reaction of metal reduction involving the evolution of H_2_, gives rise to the effect of anomalous co-deposition.(7-9) This side reaction process depletes protons and increases the concentration of OH^−^ in the electrolyte. The surge in OH^−^ leads to the formation and adsorption of metal mono-hydroxide ions or metal hydroxides X(OH)^+^ during the deposition process, which is described by:

$$2 \text{H}_{2}\text{O} + 2 e^{-} \to\text{H}_{2} + 2 \text{OH}^{-}$$

$$\text{X}^{2+}+ \text{OH}^{-}\to\text{X} (\text{OH)}^{\text{+}}$$

$$\text{X} (\text{OH)}^{\text{+}}\to\text{X} (\text{OH)}_{ \text{ads}}^{+}$$

$$\text{X} (\text{OH)}_{\text{ads}}^{+}+2 e^{-}\to\text{X} + \text{OH}^{-}$$

where X refers to Ni and Fe atoms.

**Figure S2** The graph of high potential pulse *V_H_* vs Fe% in our NiFe thin film, nanowires and MNPs.

Magnetic hyperthermia is a well-established method for treating cancerous tumors, which uses localized heat generated by the relaxation mechanism of the MNPs in a high frequency alternating magnetic field, thereby triggering cancer cell apoptosis and tumor regression.(10-13) Many theoretical papers has already reported on the various theories describing hysteresis loops of MNPs used to calculate their theoretical specific absorption rate (SAR) values for comparison to their experimental values. To maintain a low MNPs dose and short treatment duration in magnetic hyperthermia, the MNPs heating efficiency must be maximized. The measurement of SAR, is given by the heat dissipated per unit of mass of MNPs (Wg^-1^):

$$\text{SAR}=\frac{A f}{\rho}$$

where *ρ* is the density of MNPs.

**I. Linear Response Theory**

Linear response theory (LRT) describes the dynamic response of an assembly of MNPs using the Néel and Brown relaxation time which assumes a linear response of the MNPs to the magnetic field.(14-19) The alternating magnetic field is given by:

$$H(t)=H_{\max} \cos(\omega t)$$

where ω is the angular frequency of the magnetic field. The Néel–Brown relaxation time is given by:

$$t=t_{0}\exp\left(\frac{K_{\text{eff}} V}{k_{\text{B}} T}\right)$$

The hysteresis area for MNPs with random orientations is given by:

$$A=\frac{\pi\mu_{0}^{2} H_{\max}^{2}M_{s}^{2} V}{3 k_{\text{B}} T}\frac{\omega t}{1+\omega^{2} t^{2}}$$

where *µ_0_* is the permeability of the vacuum, *k_B_* is the Boltzmann constant, *M_s_* is the saturation magnetization for the bulk material, *V* is the volume of the MNPs, *T* is the absolute temperature, *K_eff_* is the effective uniaxial anisotropy constant, and *t_0_* is the frequency factor of the Néel and Brown relaxation time (10^−9^–10^−12^s). LRT is valid for MNPs with strong anisotropy under the application low magnetic fields, when$\xi=\frac{\mu_{0} H_{\max}M_{s} V}{k_{\text{B}} T }<1$. The LRT is still valid at the transition region between superparamagnetic (*ωt* < 1) and ferromagnetism (*ωt* > 1), where the hysteresis loop area is at a maximum for small amplitude magnetic fields.

**II. Stoner–Wohlfarth Theory**

Stoner–Wohlfarth model based theories (SWMBTs) omit thermal activation, which is justified at temperature T = 0 or infinite magnetic field frequency *f* → ∞. The hysteresis area for MNPs with random orientations is given by:

$$A=2 \mu_{0} H_{c} M_{s}$$

where *H_c_* is the coercive field. For finite frequencies *f* and temperatures T ≠ 0, the hysteresis area is given by:

$$A ( T, f )=2 \mu_{0} H_{c}( T, f )M_{s}$$

The analytical formula for a temperature-dependent *H_c_* based on an approximation of the measurement time *t_m_*, with anisotropy field *µ_0_H_k_* = 2*K_eff_ /M_s_*, is given by:

$$\mu_{0} H_{c}=0.48 \mu_{0} H_{k}\left(1-\left(\frac{k_{\text{B}} T}{K_{eff} V}\left(\ln\frac{t_{m}}{t_{0}}\right)\right)^{\frac{3}{4}}\right)$$

SWMBTs are more suitable for modelling the heat dissipation for randomly orientated MNPs that are far from the superparamagnetic and ferromagnetic transition region.

As the size of MNPs increases, incoherent reversal modes causes a reduction in *H_c_*, making LRT and SWMBTs invalid. The limit of validity of LRT is at ξ < 1, when *M* varies linearly with *H* and SAR ∝ H^2^. When ξ > 1, SWMBTs is valid for *µ_0_H_max_* > 2*µ_0_H_c_* and *H_c_* independent of MNP volume. Lastly, MNPs with large sizes allow the formation of magnetic vortex or multi-domains and DWs. In such cases, the magnetization reversal process occurs by DWs nucleation and propagation, as described by Rayleigh model and SAR ∝ H^3^.(19, 20)

Single Domain

Domain

*MNP size*

Magnetization Configuration

Magnetic

Phase

Model

Super-

paramagnetic Regime

Ferromagnetic

Regime

Multi-Domain

Incoherent Reversal

Rayleigh Model
SAR ∝ H^3^

LRT

ξ < 1

SAR ∝ H^2^

SWMBTs

*µ_0_H_max_* > 2*µ_0_H_c_*

**Figure S3** Illustration of the analytical models to describe the heat dissipation of MNPs, as a function of their size and magnetic phase (superparamagnetic or ferromagnetic regime).(19, 20)

**III. Hysteresis Losses**

For single domain MNPs, the theoretical model to calculate the dynamic hysteresis loop has been proposed by Carrey et al.(19) For multi-domains MNPs, the use of micromagnetic simulations to obtain static hysteresis loop for calculation was reasonable for MNPs with large sizes, above the critical size for superparamagnetism, as the switching time of the magnetization is in the order of 10^-9^s. Since the switching time of magnetic hyperthermia is in the order ~10^-6^s, the large MNPs is able to keep up with the alternating magnetic field.

In this work, the area of hysteresis loops from micromagnetic simulations of NiFe MNPs and VSM measurements was used to theoretically calculate the SAR values. In an alternating magnetic field, the heat dissipated by the MNPs in one magnetic field cycle equals the area of the hysteresis loop *A*, given by:

$$A=\int_{-H_{\max}}^{+H_{\max}} \mu_{0} M(H) dH$$

where *M*(*H*) is the magnetization of MNPs, in an alternating magnetic field with frequency *f* and amplitude *µ_0_H_max_*.(21-23)

Endocytosis is a type of active transport that enables the uptake of macromolecules and particles from the surrounding medium into a cell.(24) The plasma membrane of the cell surrounds the targeted particle forming a pocket, which then buds off inside the cell to form a newly created intracellular vesicle with the ingested particle, as shown in Figure S4. When the HeLa cells were exposed to the MNPs, some MNPs were internalized to the cells due to endocytosis, and accumulated in the digestive vacuoles. This process causes the cell to overload and leads to induced cell death.(25, 26)


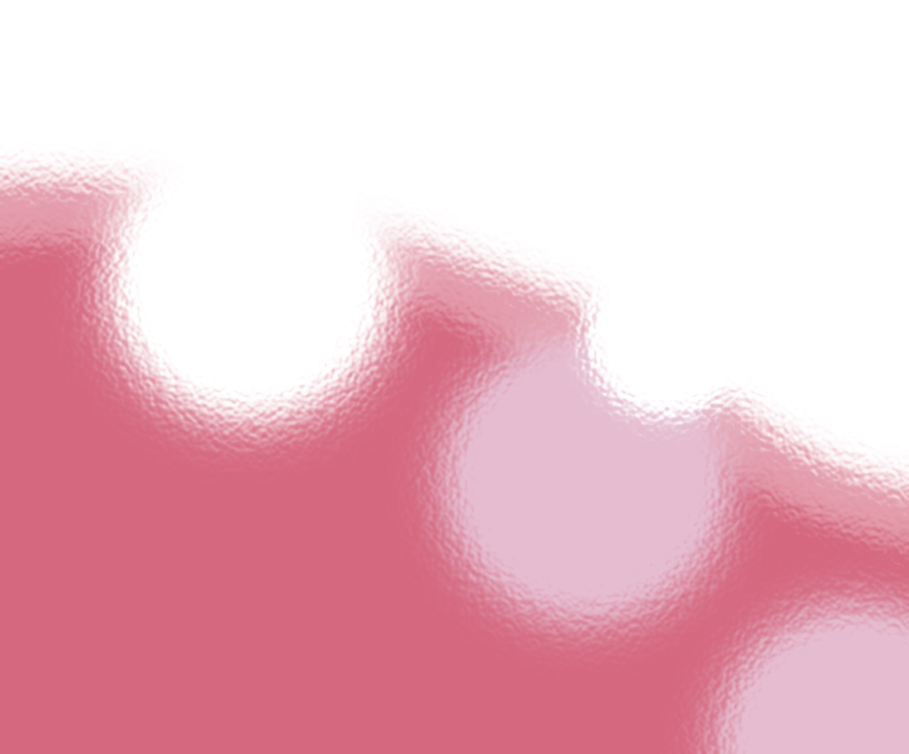

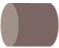

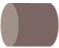

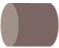

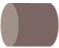

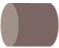

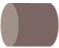

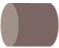

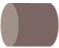

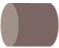

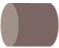

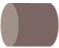

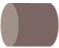

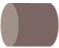

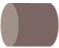

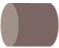

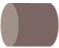

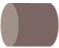


Vesicle

Cytoplasm


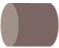


**Figure S4** Illustration of endocytosis of MNPs.

The decrease in cell viability after incubation for 48 hours was due to the MNPs being endocytosed by the HeLa cells due to weak cell adhesive interactions between the MNPs and cell membrane. The optical imaging of the HeLa cells incubated with MNPs for 48 hours without any magnetic field treatment has verified the above phenomenon of endocytosis. The cells were observed to be smaller and less spread with greater stimulated formation of lamellipodia and filopodia, as shown in Figure S5. When the MNPs are endocytosed or have lost contact with the cell surface, they are rendered ineffective for the magnetic field treatments. In addition, the cells are at an increased risk of cytotoxicity from the MNPs and effects of overloading. Surface functionalizing of MNPs with specific attachment can be explored to help prevent the MNPs from detaching from the cell surface and remain attached to cell surface receptors to reduce endocytosis and prolonged stimulation.


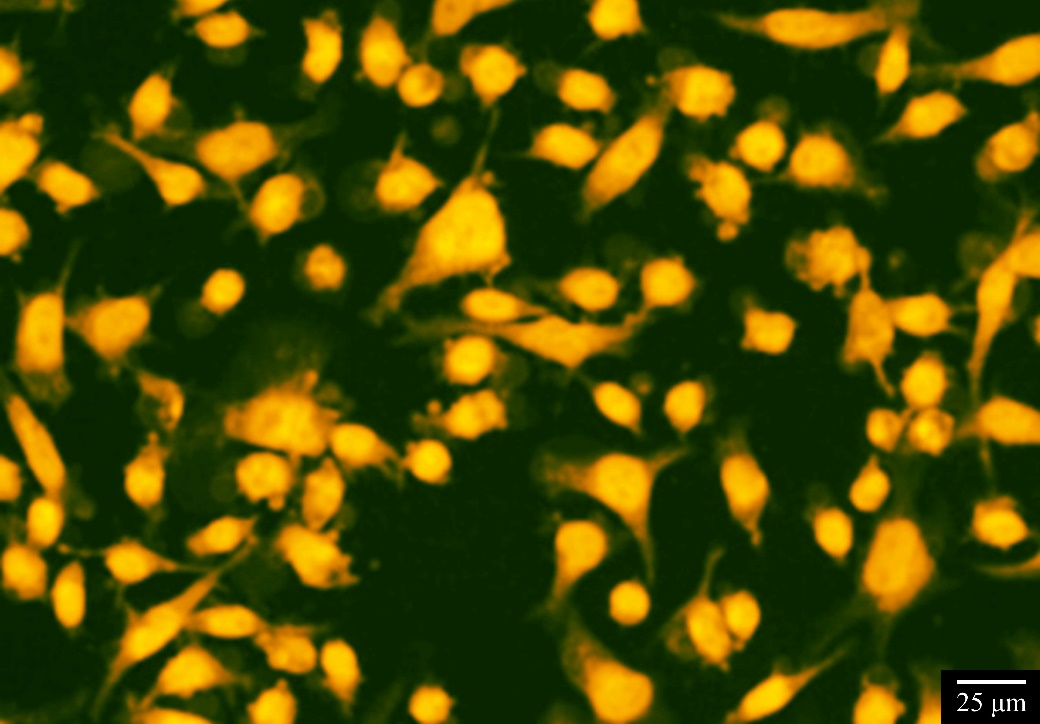


**Figure S5** Optical image of HeLa cells and NiFe MNPs incubated for 48 hours without magnetic field treatment.

**References**

1. Gan WL, Chandra Sekhar M, Wong DW, Purnama I, Chiam SY, Wong LM, et al. Multi-vortex states in magnetic nanoparticles. Applied Physics Letters. 2014;105(15):152405.

2. Salem MS, Sergelius P, Corona RM, Escrig J, Gorlitz D, Nielsch K. Magnetic properties of cylindrical diameter modulated Ni80Fe20 nanowires: interaction and coercive fields. Nanoscale. 2013;5(9):3941-7.

3. Pereira A, Palma JL, Vazquez M, Denardin JC, Escrig J. A soft/hard magnetic nanostructure based on multisegmented CoNi nanowires. Phys Chem Chem Phys. 2015;17(7):5033-8.

4. Matlosz M. Competitive Adsorption Effects in the Electrodeposition of Iron‐Nickel Alloys. Journal of the Electrochemical Society. 1993;140(8):2272-9.

5. Llavona Á, Pérez L, Sánchez MC, de Manuel V. Enhancement of anomalous codeposition in the synthesis of Fe–Ni alloys in nanopores. Electrochimica Acta. 2013;106:392-7.

6. Dragos O, Chiriac H, Lupu N, Grigoras M, Tabakovic I. Anomalous codeposition of fcc NiFe nanowires with 5–55% Fe and their morphology, crystal structure and magnetic properties. Journal of The Electrochemical Society. 2016;163(3):D83-D94.

7. Salem MS, Sergelius P, Zierold R, Moreno JMM, Görlitz D, Nielsch K. Magnetic characterization of nickel-rich NiFe nanowires grown by pulsed electrodeposition. Journal of Materials Chemistry. 2012;22(17):8549-57.

8. Matulis J, Sližys R. On some characteristics of cathodic processes in nickel electrodeposition. Electrochimica Acta. 1964;9(9):1177-88.

9. Grimmett DL, Schwartz M, Nobe K. Pulsed Electrodeposition of Iron‐Nickel Alloys. Journal of the Electrochemical Society. 1990;137(11):3414-8.

10. Golovin YI, Gribanovsky SL, Golovin DY, Klyachko NL, Majouga AG, Master capital A C, et al. Towards nanomedicines of the future: Remote magneto-mechanical actuation of nanomedicines by alternating magnetic fields. J Control Release. 2015;219:43-60.

11. Ling Y, Tang X, Wang F, Zhou X, Wang R, Deng L, et al. Highly efficient magnetic hyperthermia ablation of tumors using injectable polymethylmethacrylate–Fe3O4. RSC Adv. 2017;7(5):2913-8.

12. Zhang W, Zuo X, Niu Y, Wu C, Wang S, Guan S, et al. Novel nanoparticles with Cr3+ substituted ferrite for self-regulating temperature hyperthermia. Nanoscale. 2017;9(37):13929-37.

13. Cabrera D, Lak A, Yoshida T, Materia ME, Ortega D, Ludwig F, et al. Unraveling viscosity effects on the hysteresis losses of magnetic nanocubes. Nanoscale. 2017;9(16):5094-101.

14. Verde EL, Landi GT, Gomes JdA, Sousa MH, Bakuzis AF. Magnetic hyperthermia investigation of cobalt ferrite nanoparticles: Comparison between experiment, linear response theory, and dynamic hysteresis simulations. Journal of Applied Physics. 2012;111(12):123902.

15. Hergt R, Andra W, d'Ambly CG, Hilger I, Kaiser WA, Richter U, et al. Physical limits of hyperthermia using magnetite fine particles. IEEE Transactions on magnetics. 1998;34(5):3745-54.

16. Rosensweig RE. Heating magnetic fluid with alternating magnetic field. Journal of magnetism and magnetic materials. 2002;252:370-4.

17. Maenosono S, Saita S. Theoretical assessment of FePt nanoparticles as heating elements for magnetic hyperthermia. IEEE transactions on magnetics. 2006.

18. Sawyer CA, Habib AH, Miller K, Collier KN, Ondeck CL, McHenry ME. Modeling of temperature profile during magnetic thermotherapy for cancer treatment. Journal of Applied Physics. 2009;105(7):07B320.

19. Carrey J, Mehdaoui B, Respaud M. Simple models for dynamic hysteresis loop calculations of magnetic single-domain nanoparticles: Application to magnetic hyperthermia optimization. Journal of Applied Physics. 2011;109(8):083921.

20. Cobianchi M, Guerrini A, Avolio M, Innocenti C, Corti M, Arosio P, et al. Experimental determination of the frequency and field dependence of Specific Loss Power in Magnetic Fluid Hyperthermia. Journal of Magnetism and Magnetic Materials. 2017;444:154-60.

21. Hervault A, Thanh NTK. Magnetic nanoparticle-based therapeutic agents for thermo-chemotherapy treatment of cancer. Nanoscale. 2014;6(20):11553-73.

22. Shaterabadi Z, Nabiyouni G, Soleymani M. Physics responsible for heating efficiency and self-controlled temperature rise of magnetic nanoparticles in magnetic hyperthermia therapy. Progress in biophysics and molecular biology. 2017.

23. Delavari H, Hosseini HRM, Wolff M. Modeling of self-controlling hyperthermia based on nickel alloy ferrofluids: Proposition of new nanoparticles. Journal of Magnetism and Magnetic Materials. 2013;335:59-63.

24. Cooper G. The Cell: A Molecular Approach, 2nd edn. The Cell: A Molecular Approach. Sunderland, MA. USA: Sinauer Associates; 2000.

25. Gupta AK, Gupta M. Cytotoxicity suppression and cellular uptake enhancement of surface modified magnetic nanoparticles. Biomaterials. 2005;26(13):1565-73.

26. Gupta AK, Curtis AS. Lactoferrin and ceruloplasmin derivatized superparamagnetic iron oxide nanoparticles for targeting cell surface receptors. Biomaterials. 2004;25(15):3029-40.
